# Supplementary material for: The associations between autistic traits and disordered eating/drive for muscularity are independent of anxiety and depression in females but not males
Source: PLoS One. 2022 Oct 17;17(10):e0276249. doi: 10.1371/journal.pone.0276249 (PMC9576073; doi:10.1371/journal.pone.0276249)
Supplement: S1 Table — Six hierarchical regression models stratified by sex with DMS total score as outcome (1a and 1b), EAT-26 as outcome (1c and 1d), and DMS body image as outcome (1e and 1f). (DOCX) [file pone.0276249.s001.docx]

**Supplementary Materials**

**Supplementary Table 1a.** Hierarchical linear regression models with DMS total score as the outcome (female only sample).

|  | Step 1 | | | | |  | Step 2 | | | | |  | Step 3 | | | | |  | Step 4 | | | | |
| --- | --- | --- | --- | --- | --- | --- | --- | --- | --- | --- | --- | --- | --- | --- | --- | --- | --- | --- | --- | --- | --- | --- | --- |
|  | Unstandardised coefficients | |  | Standardised coefficients | |  | Unstandardised coefficients | |  | Standardised coefficients | |  | Unstandardised  coefficients | |  | Standardised coefficients | |  | Unstandardised  coefficients | |  | Standardised coefficients | |
|  | B | 95%CI |  | Beta | p |  | B | 95%CI |  | Beta | p |  | B | 95%CI |  | Beta | p |  | B | 95%CI |  | Beta | p |
| (Constant) | 27.754 | 26.720, 28.788 |  |  | < 0.001 |  | 35.980 | 30.507,41.453 |  |  | < 0.001 |  | 36.321 | 30.831, 41.811 |  |  | < 0.001 |  | 36.406 | 30.882, 41.930 |  |  | < 0.001 |
| AQ total score | 0.119 | 0.007, 0.230 |  | 0.104 | 0.037 |  | 0.119 | 0.009, 0.228 |  | 0.104 | 0.035 |  | 0.165 | 0.035, 0.295 |  | 0.145 | 0.013 |  | 0.175 | 0.038, 0.312 |  | 0.154 | 0.012 |
| Age |  |  |  |  |  |  | -0.165 | -0.257, -0.074 |  | -0.18*8* | < 0.001 |  | -0.163 | -0.255, -0.071 |  | -0.185 | 0.001 |  | -0.163 | -0.256, -0.070 |  | -0.185 | 0.001 |
| BMI |  |  |  |  |  |  | -0.287 | -0.514, -0.061 |  | -0.131 | 0.013 |  | -0.290 | -0.518, -0.063 |  | -0.132 | 0.012 |  | -0.229 | -0.528, -0.070 |  | -0.136 | 0.011 |
| Over-evaluation |  |  |  |  |  |  | 0.636 | 0.081, 1.191 |  | 0.120 | 0.025 |  | 0.671 | 0.090, 1.252 |  | 0.127 | 0.024 |  | 0.668 | 0.098, 1.277 |  | 0.130 | 0.022 |
| Anxiety |  |  |  |  |  |  |  |  |  |  |  |  | -0.097 | -0.373, 0.180 |  | -0.040 | 0.491 |  | -0.125 | -0.428, 0.179 |  | -0.052 | 0.420 |
| AQ*anxiety |  |  |  |  |  |  |  |  |  |  |  |  | -0.020 | -0.046, 0.006 |  | -0.079 | 0.129 |  | -0.028 | -0.060, 0.004 |  | -0.110 | 0.089 |
| Depression |  |  |  |  |  |  |  |  |  |  |  |  |  |  |  |  |  |  | 0.041 | -0.327, 0.410 |  | -0.013 | 0.825 |
| AQ*Depression |  |  |  |  |  |  |  |  |  |  |  |  |  |  |  |  |  |  | 0.017 | -0.024, 0.058 |  | 0.049 | 0.417 |
| Model fit | *F* (1, 399) = 4.395, *p* = 0.037 | | | | |  | *F* (4, 396) = 10.470, *p* < 0.001 | | | | |  | *F* (6, 394) = 7.399, *p* < 0.001 | | | | |  | *F* (8, 392) = 5.620, *p* < 0.001 | | | | |
| *R^2^* | 0.011 | | | | |  | 0.096 | | | | |  | 0.101 | | | | |  | 0.103 | | | | |
| Δ *R*^2^ |  | | | | |  | 0.085 | | | | |  | 0.006 | | | | |  | 0.002 | | | | |
| Adjusted *R*^2^ | 0.008 | | | | |  | 0.087 | | | | |  | 0.088 | | | | |  | 0.085 | | | | |
| Δ Adjusted *R*^2^ |  | | | | |  | 0.079 | | | | |  | 0.001 | | | | |  | -0.003 | | | | |
| *F* change |  | | | | |  | *F* (3, 396) = 12.369, *p* < 0.001 | | | | |  | *F* (2, 394) = 1.233, *p* = 0.293 | | | | |  | *F* (2, 392) = 0.355, *p* = 0.701 | | | | |

**Supplementary Table 1b.** Hierarchical linear regression models with DMS total score as the outcome (male only sample).

|  | Step 1 | | | | |  | Step 2 | | | | |  | Step 3 | | | | |  | Step 4 | | | | |
| --- | --- | --- | --- | --- | --- | --- | --- | --- | --- | --- | --- | --- | --- | --- | --- | --- | --- | --- | --- | --- | --- | --- | --- |
|  | Unstandardised coefficients | |  | Standardised coefficients | |  | Unstandardised coefficients | |  | Standardised coefficients | |  | Unstandardised  coefficients | |  | Standardised coefficients | |  | Unstandardised  coefficients | |  | Standardised coefficients | |
|  | B | 95%CI |  | Beta | p |  | B | 95%CI |  | Beta | p |  | B | 95%CI |  | Beta | p |  | B | 95%CI |  | Beta | p |
| (Constant) | 41.296 | 40.156, 42.436 |  |  | < 0.001 |  | 44.559 | 40.191,48.927 |  |  | < 0.001 |  | 44.799 | 40.423, 49.175 |  |  | < 0.001 |  | 44.636 | 40.223, 49.050 |  |  | < 0.001 |
| AQ total score | 0.111 | -0.039, 0.261 |  | 0.057 | 0.148 |  | 0.032 | -0.103, 0.168 |  | 0.017 | 0.639 |  | -0.020 | -0.163, 0.124 |  | -0.010 | 0.787 |  | -0.012 | -0.162, 0.139 |  | -0.006 | 0.880 |
| Age |  |  |  |  |  |  | -0.340 | -0.430, -0.250 |  | -0.268 | < 0.001 |  | -0.323 | -0.414, -0.231 |  | -0.254 | < 0.001 |  | -0.320 | -0.412, -0.229 |  | -0.252 | < 0.001 |
| BMI |  |  |  |  |  |  | -0.090 | -0.226, 0.046 |  | -0.047 | 0.196 |  | -0.082 | -0.218, 0.054 |  | -0.043 | 0.236 |  | -0.081 | -0.217, 0.056 |  | -0.043 | 0.246 |
| Over-evaluation |  |  |  |  |  |  | 2.842 | 2.258, 3.426 |  | 0.347 | < 0.001 |  | 2.646 | 2.035, 3.257 |  | 0.323 | < 0.001 |  | 2.667 | 2.051, 3.283 |  | 0.325 | < 0.001 |
| Anxiety |  |  |  |  |  |  |  |  |  |  |  |  | 0.299 | 0.024, 0.574 |  | 0.086 | 0.033 |  | 0.354 | 0.027, 0.681 |  | 0.101 | 0.034 |
| AQ*anxiety |  |  |  |  |  |  |  |  |  |  |  |  | -0.006 | -0.036, 0.024 |  | -0.014 | 0.701 |  | -0.008 | -0.048, 0.031 |  | -0.019 | 0.676 |
| Depression |  |  |  |  |  |  |  |  |  |  |  |  |  |  |  |  |  |  | -0.116 | -0.401, 0.258 |  | -0.029 | 0.542 |
| AQ*Depression |  |  |  |  |  |  |  |  |  |  |  |  |  |  |  |  |  |  | 0.004 | -0.040, 0.048 |  | 0.009 | 0.852 |
| Model fit | *F* (1, 642) = 2.101, *p* = 0.148 | | | | |  | *F* (4, 639) = 42.280, *p* < 0.001 | | | | |  | *F* (6, 637) = 29.064, *p* < 0.001 | | | | |  | *F* (8, 635) = 21.789, *p* < 0.001 | | | | |
| *R^2^* | 0.003 | | | | |  | 0.209 | | | | |  | 0.215 | | | | |  | 0.215 | | | | |
| Δ *R*^2^ |  | | | | |  | 0.206 | | | | |  | 0.006 | | | | |  | 0.000 | | | | |
| Adjusted *R*^2^ | 0.002 | | | | |  | 0.204 | | | | |  | 0.208 | | | | |  | 0.205 | | | | |
| Δ Adjusted *R*^2^ |  | | | | |  | 0.202 | | | | |  | 0.004 | | | | |  | -0.003 | | | | |
| *F* change |  | | | | |  | *F* (3, 639) = 55.495, *p* < 0.001 | | | | |  | *F* (2, 637) = 2.291, *p* = 0.102 | | | | |  | *F* (2, 635) = 0.186, *p* = 0.830 | | | | |

**Supplementary Table 1c.** Hierarchical linear regression models with EAT-26 total score as the outcome (female only sample).

|  | Step 1 | | | | |  | Step 2 | | | | |  | Step 3 | | | | |  | Step 4 | | | | |
| --- | --- | --- | --- | --- | --- | --- | --- | --- | --- | --- | --- | --- | --- | --- | --- | --- | --- | --- | --- | --- | --- | --- | --- |
|  | Unstandardised coefficients | |  | Standardised coefficients | |  | Unstandardised coefficients | |  | Standardised coefficients | |  | Unstandardised  coefficients | |  | Standardised coefficients | |  | Unstandardised  coefficients | |  | Standardised coefficients | |
|  | B | 95%CI |  | Beta | p |  | B | 95%CI |  | Beta | p |  | B | 95%CI |  | Beta | p |  | B | 95%CI |  | Beta | p |
| (Constant) | 6.690 | 3.849, 9.531 |  |  | < 0.001 |  | -0.380 | -5.752, .992 |  |  | 0.889 |  | -2.336 | -7.945, 3.274 |  |  | 0.414 |  | -2.333 | -7.939, 3.274 |  |  | 0.414 |
| AQ total score | 0.391 | 0.257, 0.526 |  | 0.274 | < 0.001 |  | 0.265 | 0.154, 0.375 |  | 0.186 | < 0.001 |  | 0.158 | 0.028, 0.288 |  | 0.110 | 0.009 |  | 0.130 | 0.007, 0.266 |  | 0.099 | 0.032 |
| Age |  |  |  |  |  |  | 0.008 | -0.084, 0.100 |  | 0.007 | 0.862 |  | 0.012 | -0.079, 0.104 |  | 0.011 | 0.792 |  | 0.022 | -0.070, 0.114 |  | 0.020 | 0.642 |
| BMI |  |  |  |  |  |  | -0.378 | -0.603, -0.153 |  | -0.140 | 0.001 |  | -0.354 | -0.578, -0.129 |  | -0.131 | 0.002 |  | -0.370 | -0.595, -0.146 |  | -0.137 | 0.001 |
| Over-evaluation |  |  |  |  |  |  | 4.064 | 3.504, 4.623 |  | 0.610 | < 0.001 |  | 3.865 | 3.284, 4.446 |  | 0.580 | < 0.001 |  | 3.797 | 3.210, 4.384 |  | 0.570 | < 0.001 |
| Anxiety |  |  |  |  |  |  |  |  |  |  |  |  | 0.366 | 0.090, 0.643 |  | 0.121 | 0.009 |  | 0.249 | -0.052, 0.549 |  | 0.082 | 0.105 |
| AQ*anxiety |  |  |  |  |  |  |  |  |  |  |  |  | 0.027 | 0.001, 0.052 |  | 0.083 | 0.043 |  | 0.025 | -0.007, 0.057 |  | 0.078 | 0.126 |
| Depression |  |  |  |  |  |  |  |  |  |  |  |  |  |  |  |  |  |  | 0.368 | 0.005, 0.731 |  | 0.096 | 0.047 |
| AQ*Depression |  |  |  |  |  |  |  |  |  |  |  |  |  |  |  |  |  |  | 0.003 | -0.038, 0.044 |  | 0.006 | 0.895 |
| Model fit | *F* (1, 401) = 32.582, *p* <0.001 | | | | |  | *F* (4, 398) = 71.192, *p* < 0.001 | | | | |  | *F* (6, 396) = 49.829, *p* < 0.001 | | | | |  | *F* (8, 394) = 38.057, *p* < 0.001 | | | | |
| *R^2^* | 0.075 | | | | |  | 0.417 | | | | |  | 0.430 | | | | |  | 0.436 | | | | |
| Δ *R*^2^ |  | | | | |  | 0.342 | | | | |  | 0.013 | | | | |  | 0.006 | | | | |
| Adjusted *R*^2^ | 0.073 | | | | |  | 0.411 | | | | |  | 0.422 | | | | |  | 0.424 | | | | |
| Δ Adjusted *R*^2^ |  | | | | |  | 0.338 | | | | |  | 0.011 | | | | |  | 0.002 | | | | |
| *F* change |  | | | | |  | *F* (3, 398) = 77.820, *p* < 0.001 | | | | |  | *F* (2, 396) = 4.558, *p* = 0.011 | | | | |  | *F* (2, 394) = 1.991, *p* = 0.138 | | | | |

**Supplementary Table 1d.** Hierarchical linear regression models with EAT-26 total score as the outcome (male only sample).

|  | Step 1 | | | | |  | Step 2 | | | | |  | Step 3 | | | | |  | Step 4 | | | | |
| --- | --- | --- | --- | --- | --- | --- | --- | --- | --- | --- | --- | --- | --- | --- | --- | --- | --- | --- | --- | --- | --- | --- | --- |
|  | Unstandardised coefficients | |  | Standardised coefficients | |  | Unstandardised coefficients | |  | Standardised coefficients | |  | Unstandardised  coefficients | |  | Standardised coefficients | |  | Unstandardised  coefficients | |  | Standardised coefficients | |
|  | B | 95%CI |  | Beta | p |  | B | 95%CI |  | Beta | p |  | B | 95%CI |  | Beta | p |  | B | 95%CI |  | Beta | p |
| (Constant) | 5.314 | 0.3518, 7.110 |  |  | < 0.001 |  | -0.846 | -3.617, 1.925 |  |  | 0.549 |  | -2.554 | -5.339, 0.292 |  |  | 0.078 |  | -2.804 | -5.672, 0.065 |  |  | 0.055 |
| AQ total score | 0.139 | 0.057, 0.221 |  | 0.130 | 0.001 |  | 0.078 | 0.003, 0.154 |  | 0.073 | 0.042 |  | 0.044 | -0.035, 0.123 |  | 0.041 | 0.277 |  | 0.061 | -0.022, 0.143 |  | 0.057 | 0.150 |
| Age |  |  |  |  |  |  | -0.024 | 09.974, 0.026 |  | -0.035 | 0.346 |  | -0.010 | -0.061, 0.040 |  | -0.015 | 0.683 |  | -0.009 | -0.059, 0.041 |  | -0.013 | 0.723 |
| BMI |  |  |  |  |  |  | 0.090 | 0.014, 0.166 |  | 0.087 | 0.020 |  | 0.101 | 0.026, 0.176 |  | 0.097 | 0.008 |  | 0.099 | 0.025, 0.174 |  | 0.095 | 0.009 |
| Over-evaluation |  |  |  |  |  |  | 1.756 | 1.430, 2.081 |  | 0.390 | < 0.001 |  | 1.571 | 1.235, 1.908 |  | 0.349 | < 0.001 |  | 1.568 | 1.230, 1.905 |  | 0.348 | < 0.001 |
| Anxiety |  |  |  |  |  |  |  |  |  |  |  |  | 0.250 | 0.099, 0.402 |  | 0.130 | 0.001 |  | 0.218 | 0.039, 0.398 |  | 0.114 | 0.017 |
| AQ*anxiety |  |  |  |  |  |  |  |  |  |  |  |  | 0.024 | 0.007, 0.041 |  | 0.101 | 0.005 |  | 0.039 | 0.017, 0.061 |  | 0.163 | <0.001 |
| Depression |  |  |  |  |  |  |  |  |  |  |  |  |  |  |  |  |  |  | 0.053 | -0.152, 0.259 |  | 0.025 | 0.610 |
| AQ*Depression |  |  |  |  |  |  |  |  |  |  |  |  |  |  |  |  |  |  | -0.026 | -0.050, -0.002 |  | -0.100 | 0.036 |
| Model fit | *F* (1, 642) = 11.074, *p* = 0.001 | | | | |  | *F* (4, 639) = 36.774, *p* < 0.001 | | | | |  | *F* (6, 637) = 28.742, *p* < 0.001 | | | | |  | *F* (8, 635) = 22.192, *p* < 0.001 | | | | |
| *R^2^* | 0.017 | | | | |  | 0.187 | | | | |  | 0.213 | | | | |  | 0.218 | | | | |
| Δ *R*^2^ |  | | | | |  | 0.170 | | | | |  | 0.026 | | | | |  | 0.005 | | | | |
| Adjusted *R*^2^ | 0.015 | | | | |  | 0.182 | | | | |  | 0.206 | | | | |  | 0.209 | | | | |
| Δ Adjusted *R*^2^ |  | | | | |  | 0.167 | | | | |  | 0.024 | | | | |  | 0.003 | | | | |
| *F* change |  | | | | |  | *F* (3, 639) = 44.589, *p* < 0.001 | | | | |  | *F* (2, 637) = 10.494, *p* < 0.001 | | | | |  | *F* (2, 635) = 2.211, *p* = 0.110 | | | | |

**Supplementary Table 1e.** Hierarchical linear regression models with DMS body image as the outcome (female only sample).

|  | Step 1 | | | | |  | Step 2 | | | | |  | Step 3 | | | | |  | Step 4 | | | | |
| --- | --- | --- | --- | --- | --- | --- | --- | --- | --- | --- | --- | --- | --- | --- | --- | --- | --- | --- | --- | --- | --- | --- | --- |
|  | Unstandardised coefficients | |  | Standardised coefficients | |  | Unstandardised coefficients | |  | Standardised coefficients | |  | Unstandardised  coefficients | |  | Standardised coefficients | |  | Unstandardised  coefficients | |  | Standardised coefficients | |
|  | B | 95%CI |  | Beta | p |  | B | 95%CI |  | Beta | p |  | B | 95%CI |  | Beta | p |  | B | 95%CI |  | Beta | p |
| (Constant) | 14.109 | 12.585, 15.633 |  |  | < 0.001 |  | 18.749 | 15.257,22.240 |  |  | < 0.001 |  | 18.721 | 15.044, 22.397 |  |  | < 0.001 |  | 18.606 | 14.924, 22.288 |  |  | < 0.001 |
| AQ total score | 0.088 | 0.016, 0.161 |  | 0.119 | 0.016 |  | 0.086 | 0.014, 0.157 |  | 0.116 | 0.019 |  | 0.109 | 0.024, 0.194 |  | 0.148 | 0.012 |  | 0.111 | 0.021, 0.200 |  | 0.149 | 0.016 |
| Age |  |  |  |  |  |  | -0.097 | -0.156, -0.037 |  | -0.170 | 0.002 |  | -0.094 | -0.154, -0.034 |  | -0.165 | 0.002 |  | -0.091 | -0.151, -0.031 |  | -0.160 | 0.003 |
| BMI |  |  |  |  |  |  | -0.164 | -0.310, -0.018 |  | -0.117 | 0.028 |  | -0.163 | -0.310, -0.016 |  | -0.116 | 0.030 |  | -0.174 | -0.321, -0.027 |  | -0.124 | 0.021 |
| Over-evaluation |  |  |  |  |  |  | 0.403 | 0.040, 0.767 |  | 0.117 | 0.030 |  | 0.404 | 0.024, 0.784 |  | 0.117 | 0.037 |  | 0.402 | 0.017, 0.787 |  | 0.116 | 0.041 |
| Anxiety |  |  |  |  |  |  |  |  |  |  |  |  | -0.029 | -0.210, 0.152 |  | -0.019 | 0.752 |  | -0.084 | -0.281, 0.113 |  | -0.053 | 0.405 |
| AQ*anxiety |  |  |  |  |  |  |  |  |  |  |  |  | -0.013 | -0.030, 0.004 |  | -0.079 | 0.130 |  | -0.020 | -0.041, 0.001 |  | -0.122 | 0.058 |
| Depression |  |  |  |  |  |  |  |  |  |  |  |  |  |  |  |  |  |  | 0.131 | -0.107, 0.368 |  | 0.066 | 0.281 |
| AQ*Depression |  |  |  |  |  |  |  |  |  |  |  |  |  |  |  |  |  |  | 0.015 | -0.012, 0.042 |  | 0.067 | 0.265 |
| Model fit | *F* (1, 401) = 5.808, *p* = 0.016 | | | | |  | *F* (4, 398) = 9.369, *p* < 0.001 | | | | |  | *F* (6, 396) = 6.635, *p* < 0.001 | | | | |  | *F* (8, 394) = 5.282, *p* < 0.001 | | | | |
| *R^2^* | 0.014 | | | | |  | 0.086 | | | | |  | 0.091 | | | | |  | 0.097 | | | | |
| Δ *R*^2^ |  | | | | |  | 0.072 | | | | |  | 0.005 | | | | |  | 0.006 | | | | |
| Adjusted *R*^2^ | 0.012 | | | | |  | 0.077 | | | | |  | 0.078 | | | | |  | 0.079 | | | | |
| Δ Adjusted *R*^2^ |  | | | | |  | 0.065 | | | | |  | 0.001 | | | | |  | 0.001 | | | | |
| *F* change |  | | | | |  | *F* (3, 398) = 10.419, *p* < 0.001 | | | | |  | *F* (2, 396) = 1.154, *p* = 0.316 | | | | |  | *F* (2, 394) = 1.201, *p* = 0.302 | | | | |

**Supplementary Table 1f.** Hierarchical linear regression models with DMS body image as the outcome (male only sample).

|  | Step 1 | | | | |  | Step 2 | | | | |  | Step 3 | | | | |  | Step 4 | | | | |
| --- | --- | --- | --- | --- | --- | --- | --- | --- | --- | --- | --- | --- | --- | --- | --- | --- | --- | --- | --- | --- | --- | --- | --- |
|  | Unstandardised coefficients | |  | Standardised coefficients | |  | Unstandardised coefficients | |  | Standardised coefficients | |  | Unstandardised  coefficients | |  | Standardised coefficients | |  | Unstandardised  coefficients | |  | Standardised coefficients | |
|  | B | 95%CI |  | Beta | p |  | B | 95%CI |  | Beta | p |  | B | 95%CI |  | Beta | p |  | B | 95%CI |  | Beta | p |
| (Constant) | 21.550 | 19.529, 23.571 |  |  | < 0.001 |  | 25.568 | 19.529,23.571 |  |  | < 0.001 |  | 23.887 | 20.817,26.957 |  |  | < 0.001 |  | 24.099 | 20.997,27.200 |  |  | < 0.001 |
| AQ total score | 0.147 | 0.055, 0.239 |  | 0.122 | 0.002 |  | 0.098 | 0.017, 0.179 |  | 0.082 | 0.018 |  | -0.039 | -0.046, 0.124 |  | 0.033 | 0.364 |  | 0.027 | -0.062, 0.116 |  | 0.023 | 0.551 |
| Age |  |  |  |  |  |  | -0.248 | -0.301, -0.194 |  | -0.316 | < 0.001 |  | -0.228 | -0.282, -0.174 |  | -0.290 | < 0.001 |  | -0.231 | -0.285, -0.176 |  | -0.294 | < 0.001 |
| BMI |  |  |  |  |  |  | -0.055 | -0.136, 0.027 |  | -0.047 | 0.188 |  | -0.046 | -0.127, 0.035 |  | -0.039 | 0.264 |  | -0.048 | -0.129, 0.033 |  | -0.041 | 0.245 |
| Over-evaluation |  |  |  |  |  |  | 1.782 | 1.432, 2.132 |  | 0.352 | < 0.001 |  | 1.556 | 1.193, 1.919 |  | 0.307 | < 0.001 |  | 1.530 | 1.165, 1.895 |  | 0.302 | < 0.001 |
| Anxiety |  |  |  |  |  |  |  |  |  |  |  |  | 0.342 | 0.179, 0.505 |  | 0.159 | < 0.001 |  | 0.275 | 0.081, 0.470 |  | 0.128 | 0.005 |
| AQ*anxiety |  |  |  |  |  |  |  |  |  |  |  |  | -0.005 | -0.023, 0.013 |  | -0.018 | 0.603 |  | -0.003 | -0.027, 0.020 |  | -0.012 | 0.788 |
| Depression |  |  |  |  |  |  |  |  |  |  |  |  |  |  |  |  |  |  | -0.143 | -0.079, 0.365 |  | -0.059 | 0.206 |
| AQ*Depression |  |  |  |  |  |  |  |  |  |  |  |  |  |  |  |  |  |  | -0.002 | -0.028, 0.023 |  | -0.009 | 0.850 |
| Model fit | *F* (1, 642) = 9.773, *p* = 0.002 | | | | |  | *F* (4, 639) = 55.007, *p* < 0.001 | | | | |  | *F* (6, 637) = 40.355, *p* < 0.001 | | | | |  | *F* (8, 635) = 30.452, *p* < 0.001 | | | | |
| *R^2^* | 0.015 | | | | |  | 0.256 | | | | |  | 0.275 | | | | |  | 0.277 | | | | |
| Δ *R*^2^ |  | | | | |  | 0.241 | | | | |  | 0.019 | | | | |  | 0.002 | | | | |
| Adjusted *R*^2^ | 0.013 | | | | |  | 0.251 | | | | |  | 0.269 | | | | |  | 0.268 | | | | |
| Δ Adjusted *R*^2^ |  | | | | |  | 0.238 | | | | |  | 0.018 | | | | |  | -0.001 | | | | |
| *F* change |  | | | | |  | *F* (3, 639) = 69.049, *p* < 0.001 | | | | |  | *F* (2, 637) = 8.476, *p* <0.001 | | | | |  | *F* (2, 635) = 0.815, *p* = 0.443 | | | | |
