# Supplementary material for: The associations between autistic traits and disordered eating/drive for muscularity are independent of anxiety and depression in females but not males
Source: PLoS One. 2022 Oct 17;17(10):e0276249. doi: 10.1371/journal.pone.0276249 (PMC9576073; doi:10.1371/journal.pone.0276249)
Supplement: Attachment — Submitted filename: Plos One Response to Reviews.docx [file pone.0276249.s002.docx]

Dear Reviewers,

Thank you very much for taking the time to review our paper and providing these excellent comments to help us improve the manuscript. The comments are fair and constructive, and we appreciate your input.

Responses to each comment are provided below. The changes in the manuscript are highlighted with red text.

All the best,

Corresponding Author

**Editor**

- In response to the comments by the Editor we have now included information regarding the statistical assumptions for the regression models in the data analysis section of the method. We have also amended the results to include the numbers for adjusted *R*^2^.

**Reviewer #1**

The paper reports an exciting study on the relationships between AQ and ED psychopathology, looking for male/female differences. The authors have included a large sample of participants. They are also aware of the need to validate the online responses received, increasing their robustness. I have only a few comments for the authors to increase their clarity.

- Thank you for reviewing the work and for these valuable comments.

- in the introduction I think the authors should increase their description of connections between MD and AQ because they are not clear to me.

- We have now included a new section in the introduction labelled “rationale for the current investigation” and within this section we have increased our description of connections between MD and AQ (please see end of page 6 + page 7).

- please be aware of the use of gender or sex definition. In some parts of the paper, you use "sex/gender", but they are not the same. Moreover, it is not clear to me if you evaluate this aspect and how. Finally, sexual orientation is a key element for body weight dissatisfaction and body image concerns. This could be a bias in your analysis because a different rate between heterosexual and bisexual or gay/lesbian participants could unbalance the results (see <https://doi.org/10.1007/s40519-020-01047-7>).

- In the questionnaire we specifically asked the participants to report their sex. We have therefore changed the terminology in the paper to reflect this. We did not measure sexual orientation and therefore have included this point in the limitations section of the discussion and cited the suggested paper.

- body image concerns seem to be linked to cognitive evaluation and judgments (see <https://doi.org/10.1002/erv.2812>). I think this aspect has a role in your study due to the connections between AQ traits and executive functions and the ability to interpret judgments. Please, consider this aspect in your discussion.

- As we do not measure executive functions or judgements in this study this aspect does not form part of our discussion. We feel it would take the findings of the study too far to speculate on this aspect considering we only have correlational data on the global measures of AQ, ED and DMS. However, we agree this is relevant background information regarding the rationale for focusing on autistic traits and have therefore included it as a point in the introduction section (paragraph 7) and cited the suggested paper.

- in the discussion, the authors have poorly addressed the differences between males and females. I think this is an interesting aspect that needs more space, especially for the discussed literature about the presence of autistic traits in males and ED psychopathology in women.

- We have now added some additional literature/information in this regard in the discussion.

- While methods are clear and pre-registered, I think they should use more conservative p-values due to the presence of a very large number of analyses. Their p-values are reported al <.001 but with a large number of comparisons, a corrected p-value could be also lower. Please consider this aspect.

- We understand this concern but feel that in this case correcting for the number of tests may confuse the interpretation of results and increase the risk of a type II error. Although for completeness the tables in the paper report the statistics for all the variables (including subscale scores), and we have also conducted some additional exploratory analyses, please note that the main hypotheses as per our pre-registration plan involved only n=9 tests. To be more specific: tests 1-3 compared males and females on AQ score, EAT-26 score, and DMS score; tests 4 and 5 involved partial correlations between AQ score and EAT-26 score and AQ score and DMS score; tests 6 and 7 were Fisher r-to-z tests to examine sex differences between males and females on the aforementioned correlations; and finally, tests 8 and 9 included the two multiple regression analyses (one with EAT-26 score and the other DMS score as the outcome).

**Reviewer #2**

Dear Editor, first of all thank you for giving me the opportunity to review this interesting proposal.
The manuscript “Associations between autistic traits and disordered eating/drive for muscularity: A replication and extension of Barnett et al. (2021)” is well organized and well written. The paper shed some like on the link between autism and eating symptoms, in particular drive for muscularity. It has the strength of emphasizing differences in sex among.

- Thank you for reviewing the work and for your valuable comments.

I suggest some minor revision:
1) For esthetic sake, I suggest that the title do not include the reference with the data. I suggest rephrasing the title.

- We have now rephrased the title and removed the reference. It now reads: The associations between autistic traits and disordered eating/drive for muscularity are independent of anxiety and depression in females but not males.

2) The abstract needs to be rewritten, avoiding the numbered list, controlling spaces and phrases. Furthermore the last sentence: “The findings have both research and clinical implications” is too vague and need to be specified in some way.

- We have removed the numbered list, re-worded some of the text, and provided more specificity as suggested.

3) Results consider anxiety and depression as confounding factors but in the introductions those two conditions are not addressed properly. I suggest to add a paragraph and some references as a rationale for the analysis.

- We have added a paragraph in the introduction that explains the rationale for including anxiety and depression as confounding factors (see paragraph 8 of the introduction).

4) Among the limitations of the paper should be described the fact that the sample is not a clinical population and that no diagnosis by experienced clinician has been conducted. Authors choose not to have exclusion criteria like having an actual diagnosis of ed. This should be addressed in the limitation.

- We have now added these points into the limitations section.
